# Perforated Meckel’s diverticulum misdiagnosed as a urinary tract infection in an 11-year-old adolescent: case report of a rare differential diagnosis

**DOI:** 10.1186/s13037-025-00443-1

**Published:** 2025-07-03

**Authors:** Mohamed Ali, Hisham Hazem Warda, Ahmed Elghrieb

**Affiliations:** 1 Health Affairs Directorate – Dakahlia, Mansoura, Egypt; 2 Department of General Surgery, Faculty of Medicine, Mansoura University, Mansoura, Egypt

**Keywords:** Meckel’s diverticulum, Urinary tract infection, Laparoscopic diverticulectomy, Differential diagnosis

## Abstract

**Background:**

Meckel’s diverticulum, a congenital anomaly of the gastrointestinal tract, is often asymptomatic but can present with complications such as inflammation, perforation, or obstruction. Misdiagnosis is common owing to its varied presentations, particularly when symptoms mimic other conditions such as urinary tract infections (UTI).

**Case presentation:**

An 11-year-old boy presented with persistent suprapubic pain and dysuria for one week. Initial urine analysis revealed turbid urine with high numbers of red blood cells, leading to a diagnosis of urinary tract infection (UTI), and antibiotic treatment was initiated. However, the patient’s symptoms persisted, with worsening clinical signs. A complete blood count revealed leukocytosis with neutrophilia, suggesting the need for further evaluation. A non contrast computed tomography (CT) scan revealed a thickened, blind-ended structure in the midline lower abdomen with gas, extensive fat stranding, and associated mesenteric lymphadenopathy, suggestive of perforated Meckel’s diverticulum. The patient underwent laparoscopic exploration, which revealed an abscess caused by perforated Meckel’s diverticulum adherent to the urinary bladder. Diverticulectomy and incidental appendectomy were performed via a stapling device. Pathology confirmed a perforation of Meckel’s diverticulum with serofibrinous peritonitis and follicular appendicitis. The postoperative course was uneventful, with the patient resuming full oral intake by the fifth day and being discharged in stable condition.

**Conclusion:**

This case emphasizes how Meckel’s diverticulum can mimic a urinary tract infection, especially in pediatric patients with overlapping symptoms like suprapubic pain and dysuria. The delayed diagnosis highlights the importance of reconsidering rare causes when symptoms persist. Timely imaging was crucial in guiding effective treatment.

## Background

Meckel’s diverticulum is the most common congenital anomaly of the gastrointestinal tract, occurring in approximately 2% of the general population [1]. It results from incomplete obliteration of the vitelline duct during fetal development [1]. While typically asymptomatic, Meckel’s diverticulum can lead to complications such as gastrointestinal bleeding, bowel obstruction, diverticulitis, or perforation [1, 2]. These complications, although rare, often present with nonspecific symptoms that mimic other abdominal pathologies, making accurate diagnosis challenging [1, 3].

Urinary tract infection (UTI), on the other hand, are among the most common infections worldwide, particularly in women, with a prevalence of 50–60% experiencing at least one episode during their lifetime [4].

In both males and females, urinary tract infection (UTI) can present with symptoms such as abdominal or pelvic pain, urinary urgency, and dysuria, which may overlap with the clinical manifestations of other intra-abdominal conditions [5]. This overlap can lead to diagnostic confusion, particularly in cases of less common conditions such as Meckel’s diverticulum [5].

In this report, we present a unique case of perforated Meckel’s diverticulum in an 11-year-old boy that was initially misdiagnosed as a urinary tract infection (UTI) due to shared clinical features. This case highlights the diagnostic challenges posed by atypical presentations of Meckel’s diverticulum and underscores the importance of maintaining a broad differential diagnosis in patients presenting with abdominal pain and urinary symptoms.

## Case presentation

An 11-year-old boy presented to our clinic with persistent suprapubic pain lasting one week. He also experienced urinary symptoms, such as dysuria. He had no significant medical or surgical history. His vital signs included tachycardia at 120 beats per minute and a fever of 38 °C.

Urinalysis revealed turbid urine with elevated red blood cells (30–35 per high-power field; reference: 0.0–10.0), pus cells (20 per high-power field; reference: 6.0–8.0), and ketone bodies (++; reference: nil) have also been reported.

He was diagnosed with urinary tract infection (UTI) and treated with antibiotics (cefixime (Suprax) syrup 240 mg once daily) over three days for a diagnosis of cystitis. However, there was no improvement in the patient’s symptoms, and his condition deteriorated.

Three days later, a complete blood count was ordered, revealing a normal hemoglobin level of 12.7 g/dL (reference: 12.0–16.0), platelet count of 327 × 10³/µL (150.0-450.0), and high white blood cell count of 15.3 10³/µL (4.5–11.0), with a high neutrophil percentage of 76.4% (37.0–75.0) and decreased monocyte count.

**Fig. 1 and 2:**
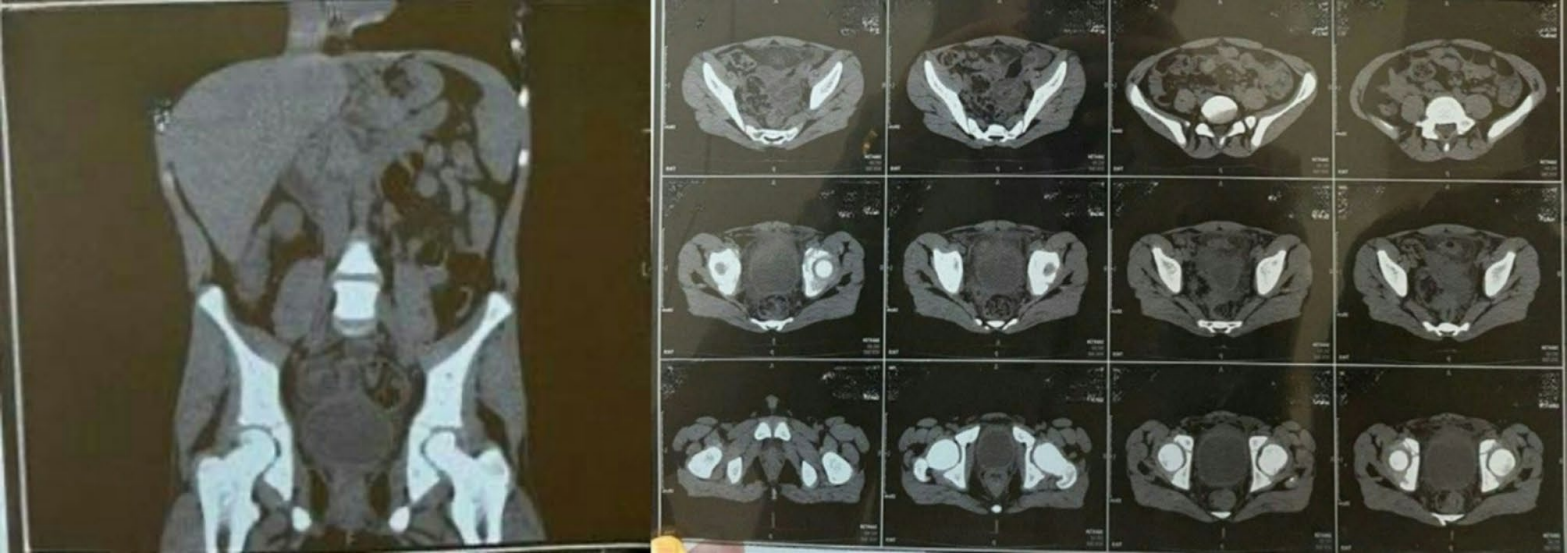
Non-contrast spiral (multi-slice) computed tomography (CT) scan of the abdomen and pelvis revealed a thickened blind-ended structure in the midline of the lower abdomen anterior to urinary bladder It shows a gas focus inside with extensive stranding of surrounding fat planes

The next day, a non-contrast spiral (multi-slice) computed tomography (CT) scan of the abdomen and pelvis was performed, which revealed the following (Figs. 1 and 2):

- A thickened, blind-ended structure was observed midline in the lower abdomen, anterior to the urinary bladder. The figure shows the gas focus inside. It is associated with extensive stranding of surrounding fat planes. It seems to be intestinal in origin… likely Meckel’s diverticulitis…for clinical correlation.
- Mural thickening of the adjacent loops is noted.
- Normal thickness of the appendix. Intact surrounding fat planes.
- Multiple mildly enlarged reactive mesenteric lymph nodes were observed.

**Fig. 3:**
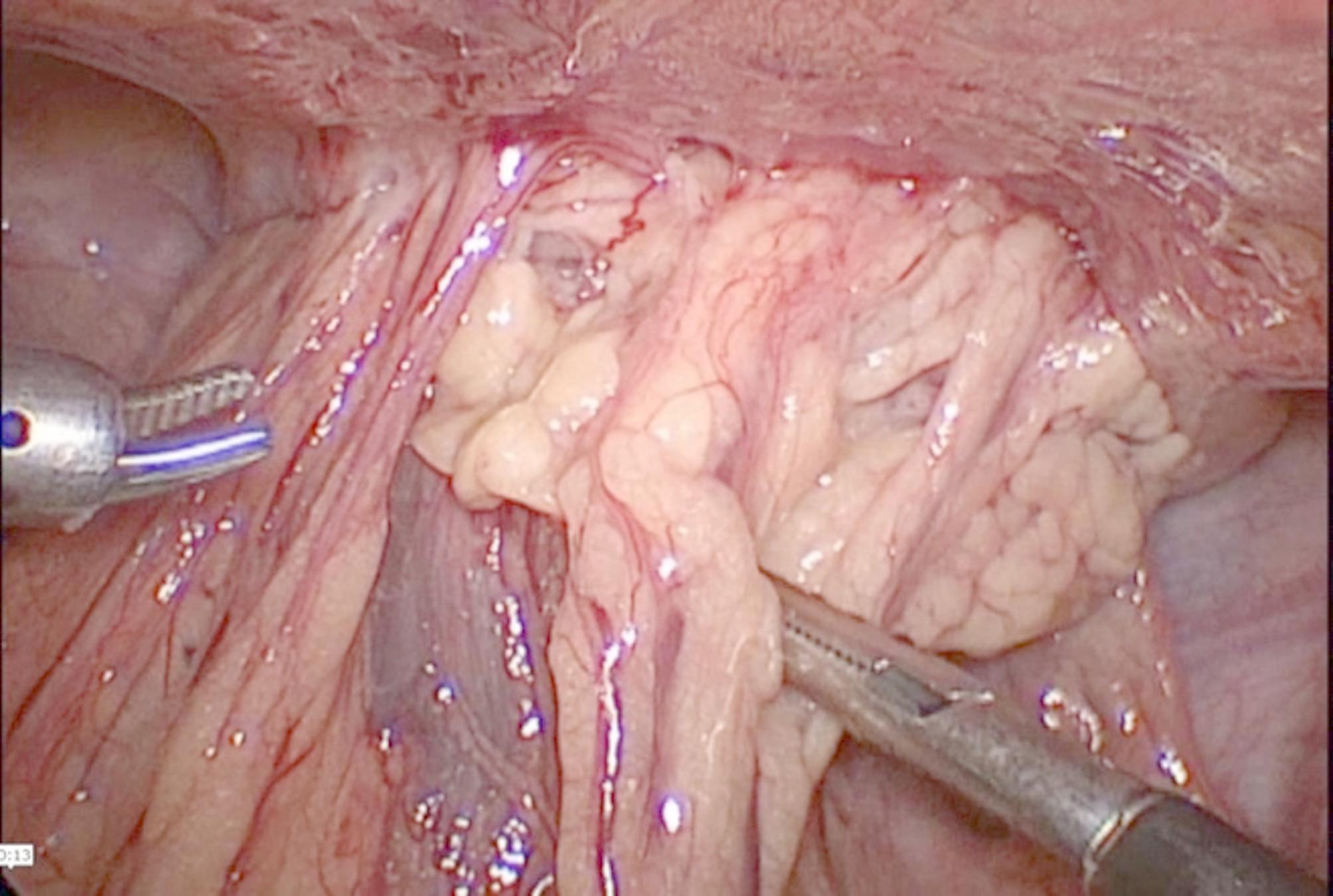
The omentum surrounding the perforated Meckel’s diverticulum

Therefore, Meckel’s diverticulum was suspected, entero-vesical fistula was also suspected but less likely as there was no air in the urinary bladder, so surgical intervention was needed. Preoperative assessments and laboratory tests were within normal ranges. Laparoscopic exploration was performed under general anesthesia with the patient in the supine position. The laparoscope tower was positioned at the surgeon, assistant, and scrub nurse stationed on the left side. We introduced a 10 mm port supra-umbilical for the laparoscope (KARL STORZ GmbH & Co. KG, Mittelstr, Tuttlingen, Germany), a 12 mm right para rectal port, and a 5 mm port between the left anterior superior iliac spine and the umbilicus were placed. Laparoscopic exploration was performed. The appendix was normal, and there was a midline mass adherent to the urinary bladder. After dissection, the mass was found to be an abscess formed by a perforated Meckel’s diverticulum surrounded by the omentum (Fig. 3).

**Fig. 4:**
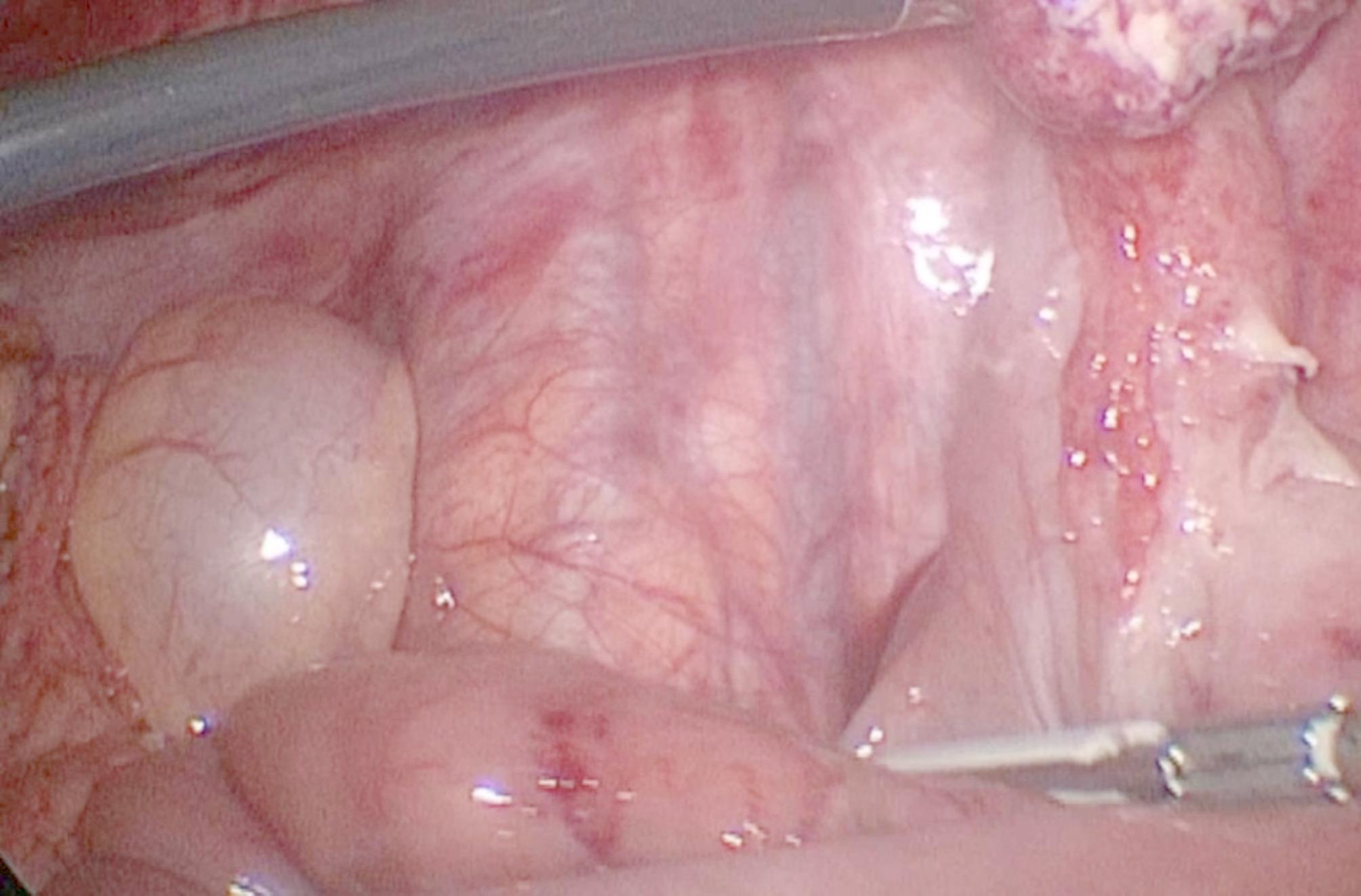
Resected Meckel’s diverticulum

After complete adhesiolysis and suction of the pus and free fluid, diverticulectomy was performed via an ECHELON FLEX™ ENDOPATH^®^ Stapler with a blue cartridge [Figure 4]. An appendectomy was also conducted as we do it routinely if acute appendicitis was one of the differential diagnoses, and the mesoappendix was dissected via a Ligassure device. Additionally, we controlled the base with intracorporeal sutures via Vicryl 0.

**Fig. 5:**
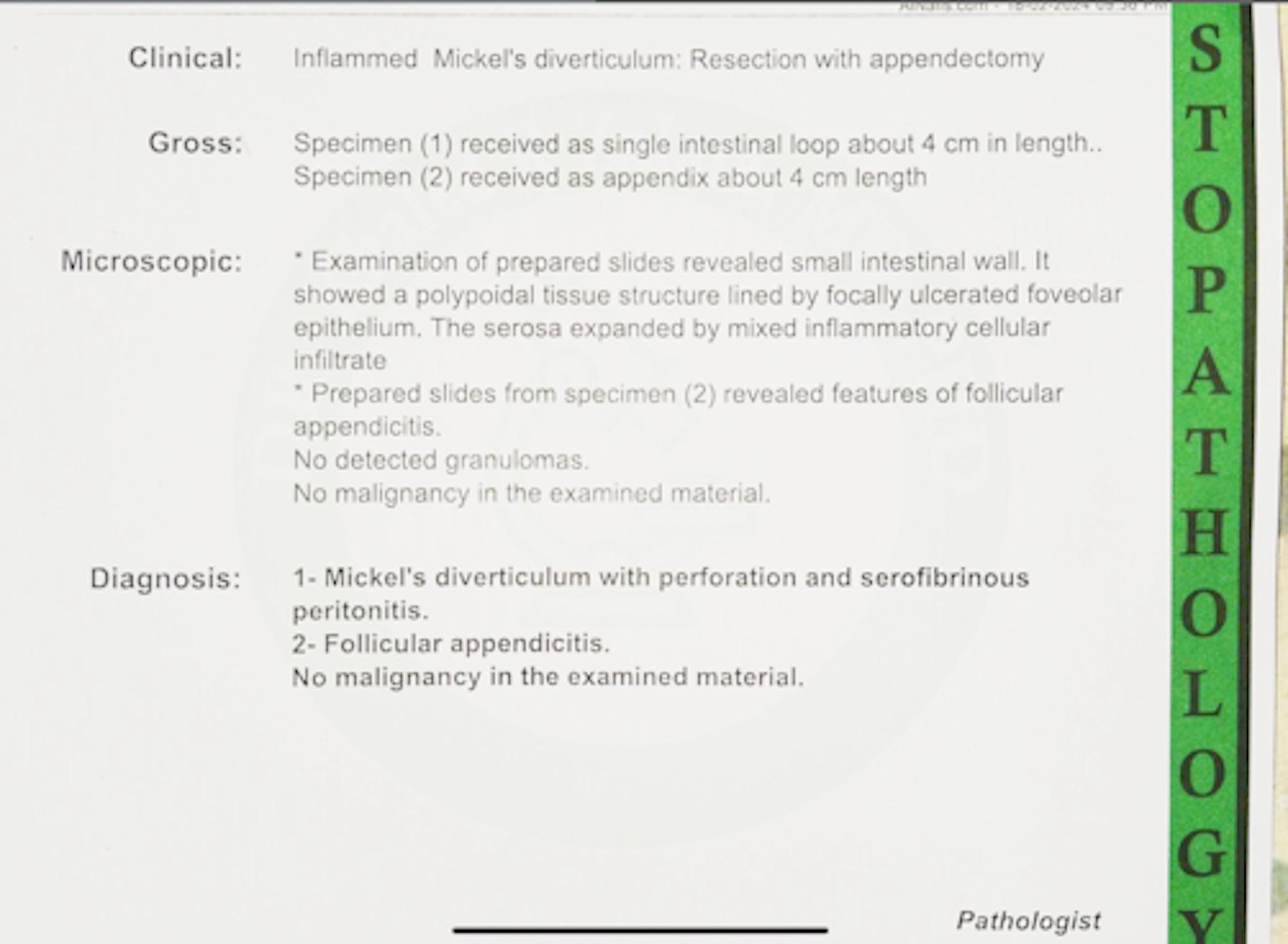
Histopathological examination confirmed a Meckel’s diverticulum with perforation and serofibrinous peritonitis. In addition to follicular appendicitis

Histopathological examination confirmed a perforated Meckel’s diverticulum with serofibrinous peritonitis and follicular appendicitis. (Fig. 5)

We introduced a sterile glove sutured from the base as a substitute for the endobag, and we placed the specimen in the bag. The bag was retrieved through a 12 mm port. A 20 French drain was inserted into the pelvis through a 5 mm port. The abdomen was deflated, and the ports were extracted under vision. The linea alba at the umbilical port was closed with Vicryl 2. Skin closure using proline 3.0 via simple sutures was performed.

Postoperatively, the patient remained nil per os for 48 h, started oral intake on the third postoperative day, and tolerated a full diet by the fifth day. The postoperative course was uneventful. There was no fever, and the patient passed motion after 24 h. The drain was removed on the 5th postoperative day, and the sutures were removed on the tenth day.

## Discussion

The persistence of ductal communication between the intestine and the yolk sac beyond the embryonic stage may result in several anomalies of the vitelline duct. These anomalies include Meckel’s diverticulum, vitelline cysts, umbilical-intestinal fistulas, and omphalomesenteric bands [6].

Meckel’s diverticulum is the most common congenital anomaly in the gastrointestinal tract [1]. This occurs due to the persistence of the proximal part of the congenital vitello-intestinal duct, and most studies suggest an incidence of between 0.6% and 4% [7]. Although many anatomical variations exist and are often discovered incidentally during evaluations performed for other reasons, it is also the most common cause of lower gastrointestinal bleeding in the pediatric age group [7]. It is a true diverticulum that is typically located on the anti-mesenteric border and contains all three coats of the intestinal wall, with its separate blood supply from the vitelline artery [7].

The incidence is equal in males and females, but complications occur more frequently in males [2, 8–11]. It is usually an asymptomatic condition, and if patients develop symptoms, they usually present with painless rectal bleeding in the first 10 years of life, with an average age of 2.5 years. [3] Rectal bleeding is often described as currant jelly or the color of brick. Children typically present with the classic “currant jelly” color, whereas adults typically present with melena, and the bleeding usually resolves without intervention (2). Complications can occur in up to 6.4% of patients [1]. These are more common in the pediatric population and mainly involve bowel obstruction with or without intussusception, gastrointestinal hemorrhage, diverticulitis and inflammation, and Littre hernias (hernia involving the bowel segment bearing Meckel’s syndrome) [1, 11].

However, diverticulitis mimicking urinary tract infection in children has not been reported.

Diverticulitis mimicking peri umbilical cellulitis in children has been reported. One patient was diagnosed with diverticulitis with suppurative inflammatory fluid collection in the lumen, and the tip of Meckel’s diverticulum was adherent to the abdominal wall around the umbilicus because of inflammation [12].

Suprapubic pain in children can be diagnostically challenging, as it overlaps with many potential conditions [5, 13]. While a urinary tract infection is often the first thing we consider—especially when there are urinary symptoms—it’s important to remember that other issues can present in a similar way [1]. Gastrointestinal causes like appendicitis, constipation, or even Meckel’s diverticulitis, as in this case, can mimic a UTI [1, 5]. In girls, gynecological causes should also be considered, such as ovarian torsion or menstrual-related pain [13]. When a patient isn’t improving as expected, we need to keep an open mind and revisit the diagnosis. A broad differential and timely imaging can make all the difference in catching less obvious conditions early.

The diagnostic criteria for Meckel’s diverticulum include the classic rule of 2s, characterized by the following: 2 inches in length, May present before the age of 2, Male-to-female ratio approximately 2:1,2 feet away from the ileocecal valve, occurring in 2% of the population, and containing 2 types of heterotopic mucosa (gastric and pancreatic) [1] anemia or gastrointestinal bleeding are the most frequent clinical manifestations and are found in up to 80% of patients [10]. This usually leads to upper and lower gastrointestinal endoscopy, where the cause of the bleeding cannot be found. The clinical manifestations that can occur are abdominal pain (68%) and intussusception (39%) [14].

Risk factors for an increased risk of developing symptoms include age younger than 50 years, male sex, a diverticulum greater than 2 cm in length, the presence of ectopic tissue, a broad-based diverticulum, and fibrous bands attached to the diverticulum (2).

Preoperative diagnosis of symptomatic Meckel’s diverticulum remains a significant challenge [15]. This is particularly true in patients who present with symptoms other than bleeding. In a study of 776 patients by Kusumoto et al., 88% of patients presenting with bleeding had a correct preoperative diagnosis versus 11% with symptoms other than bleeding [16]. The preoperative diagnosis of Meckel’s diverticulum is still an outstanding challenge—we often encounter cases that are misdiagnosed or not diagnosed preoperatively. In doubtful cases, laparoscopy is a preferred diagnostic modality [17]. However, technetium-99 m pertechnate scanning is the most common and accurate noninvasive method performed for these patients [18].

For diagnostic tools, abdominal ultrasonography can contribute to the diagnosis but often yields nonspecific findings, such as a thickened intestinal wall, fluid fille target or distended loops of the bowel [19, 20].

One of the most useful tools is computed tomography (CT) scan, which usually shows a thickened small intestinal wall, with an elongated, intraluminal, fat-attenuating lesion [21], as in our case. In the case of intussusception, a computed tomography (CT) scan is especially useful, as it can reveal the characteristic “target sign”. Inverted Meckel’s diverticulum is sometimes confused with a lipoma on computed tomography (CT) scan because it also consists of macroscopic fatty tissue. However, in most cases, abdominal computed tomography (CT) scan provides useful information for the diagnosis and treatment of inverted Meckel’s diverticulum [22].

Capsule endoscopy has recently been considered a useful diagnostic tool for diagnosing Meckel’s diverticulum [23–25]. However, the role of capsule endoscopy in the identification of Meckel’s diverticulum is not yet clear, with only a few case reports and case series available. Furthermore, in the case of inverted Meckel’s diverticulum, the information is very limited, represented by only 2 case reports [26, 27]. The capsule findings compatible with inverted Meckel’s diverticulum were described as elevated lesions with normal mucosa [26] or as pedunculated polyps [27].

**Table 1:** The following is a collection of mesenteric meckel’s diverticulum cases reported in the surgical literature. The following table was extracted from a table previously created in a previous study [28], and it was mentioned in another study [29]. In addition, 4 extra cases reported recently [30, 31, 32, 29] list the reported cases in summary (Table 1)

No.	Author/Yr	Presentation	Preop Diagnosis	Procedure	Operative Finding	Histopathology
1	Current case Mohamed Ali	11-year-old boy presented with suprapubic pain and dysuria	Urinary tract infection (UTI)	Diverticulectomy and incidental appendectomy	abscess formed by perforated mickel’s diverticulum adherent to the urinary bladder and Surrounded by the omentum	Meckel’s diverticulum with perforation and serofibrinous peritonitis In addition to Follicular appendicitis.
2	Basem AlShareef [29]	70-year-old male with left groin swelling	strangulated inguinal hernia	Segmental bowel resection + side-to-side anastomosis	Perforated mesenteric Meckel’s diverticulum + anti-mesenteric Meckel’s diverticulum	Perforated Meckel’s diverticulum associated with dense inflammation and ischemia
3	Das et al. [33]	32 years old male with lower abdominal pain	infected collection post umbilical piercing	Small bowel resection + end-to-end anastomosis	Perforated mesenteric Meckel’s diverticulum	Meckel’s diverticulum; gastric mucosa
4	Melissa et al. [31]	27 years old male with right lower quadrant pain	Acute appendicitis	Small bowel resection + anastomosis	Perforated mesenteric Meckel’s diverticulum	gastric and pancreatic tissue
5	Al-Qahtani et al. [34]	26 years old female with right lower quadrant pain	Acute appendicitis	Diverticuloectomy	Perforated mesenteric Meckel’s diverticulum	Meckel’s diverticulum with Diverticulitis
6	Abbas et al. [28]	45 years old female with abdominal pain	Chronic abdominal pain	Segmental bowel resection + end-to-end anastomosis	Mesenteric Meckel’s diverticulum	Mesenteric Meckel’s diverticulum with no malignancy nor ectopic mucosa
7	Toure et al. [35]	45 years old male with epigastric pain	Acute abdomen	Segmental resection + anastomosis	Perforated mesenteric Meckel’s diverticulum	Perforated diverticulitis without heterotopic tissue
8	Mohanty et al. [36]	16 months old male with rectal bleeding	Bleeding Meckel’s diverticulum	Small bowel resection + anastomosis	Mesenteric Meckel’s diverticulum	Antral type gastric mucosa
9	Ahmad et al. [37]	25 years old male with right lower quadrant pain	Small bowel mass	Segmental resection + anastomosis	Mesenteric mass	Chronic inflammation, no heterotopic tissue
10	Singh [38]	2 years old with umbilical serous discharge 3 years old with painless rectal bleeding with umbilical pain	--	1 + 2 + 3 = Small bowel resection + anastomosis	1 + 2 + 3 = Mesenteric diverticulum	1 = absent gastric mucosa 2 + 3 = Ectopic gastric mucosa
11	Carpenter et al. [39]	35 years old male with black stool	Meckel’s diverticulum	Small bowel resection + side-to-side anastomosis	Mesenteric diverticulum	Ectopic gastric mucosa
12	Karaman et al. [40]	23 years old male with right lower quadrant pain		Appendectomy + diverticulectomy	Inflamed Mesenteric diverticulum	Acute appendicitis with Mesenteric diverticulum
13	Seitun et al. [41]	65 years old female with right lower quadrant pain	Meckel’s diverticulum	Appendectomy + diverticulectomy	Inflammatory changes of distal ileum and diverticulum + mesenteric abscess	Severe acute transmural phlegmonous inflammation; focal area of heterotopic gastric mucosa within the head of the diverticulum with perforation
14	WalczaK et al. [35]	25 years old male with incidental finding on ultrasound	Hypogastric/mesenteric cyst	Small bowel resection + side-to-side anastomosis	Adherent mesenteric cyst	Ectopic gastric mucosa + inflammatory changes
15	Manukyan et al. [42]	15 years old female with abdominal pain	Acute abdomen	Ileal segmental resection	Perforated mesenteric diverticulum and pus and intestinal content in pelvis	Pancreatic tissue + oxyntic and antral type gastric mucosa
16	Buke et al. [43]	8 years old male with Painless rectal bleeding	Meckel’s diverticulum	Segmental resection + anastomosis	Inflamed diverticulum and lymphadenopathy	Heterotopic gastric mucosa
17	Segal et al. [44]	19 years old male with diffuse abdominal pain	Acute appendicitis	Small bowel resection + wide local excision of mesentery + appendectomy	Mesenteric mass, mesenteric thickening, adenopathy	Ectopic gastric mucosa + inflammatory changes
18	Qi Zhang [26]	37 years old male with lower abdominal pain for 2 h	acute appendicitis,	partial resection of the small intestine followed by intestinal anastomosis	Meckel’s diverticulum with internal hernia and intestinal necrosis.	small intestine diverticulum with necrosis
19	Park JS. [27]	3 years old female with acute colicky abdominal pain, vomiting and periumbilical erythema	Periumbilical Cellulitis	Segmental bowel resection and reanastomosis	long Meckel’s diverticulum with necrotic and fluid-filled cystic end	ectopic gastric mucosa was associated with ulceration and serositis
20	Ioana Anca Stefanopol [19]	16years old girl with fever (39 °C), median sub umbilical pain and dysuria.	Infected Urachal Cyst	segmental resection anastomosis	Meckel diverticulitis	acute inflammatory tissue without malignant or ectopic mucosal cells

The table summarizes how often, although the total number of cases is small, mesenteric Meckel’s diverticulum cases are misdiagnosed, as they present with a variety of nonspecific symptoms. However, once intraoperatively encountered, patients with mesenteric Meckel’s diverticulum underwent similar approaches for treatment.

Surgery is the treatment of choice for symptomatic Meckel’s diverticulum, and the traditional procedure is open diverticulectomy or segmental bowel resection and anastomosis, depending on the length of the Meckel diverticulum and the location of the ectopic mucosa [45, 46]. The general consensus is that it should be treated with resection [45, 46]. In the case of asymptomatic Meckel’s diverticulum, there is some debate. Resection is generally recommended for patients younger than 40 years, with a diverticulum longer than 2 cm, diverticula with narrow necks, fibrous bands, and/or ectopic gastric tissue, and/or when the diverticulum appears thickened and inflamed [12–47].

## Conclusion

This case illustrates Meckel’s diverticulum can be easily confused with urinary tract infection, especially when the symptoms overlap, such as in our young male patient with suprapubic pain and dysuria. The delay in diagnosis reminds us how important it is to keep an open mind and consider less common conditions when patients don’t improve as expected. Imaging played a key role in uncovering the real cause and guiding timely surgical treatment. By sharing this case, we hope to encourage clinicians to think beyond the obvious when faced with recurrent or unusual presentations.

## Data Availability

No datasets were generated or analysed during the current study.

## Abbreviations

UTI: Urinary tract infection
CT: Computed tomography
